# Retraction of duplicate publication

**Published:** 2013-01

Recently, we were made aware of a case of duplicate publication.^1,2^ A technical tip published in the *Annals* in 2009 had been published previously in *BMJ Case Reports*. Our enquiries, conducted jointly with *BMJ Case Reports*, established that the corresponding author had submitted the same material to the *Annals* immediately after it had been accepted for online publication in *BMJ Case Reports*. The text and authorship of the two publications are identical. Duplicate publication of any sort is publication malpractice, which wastes resources, including the time of editors and reviewers, as well as publication space that could be offered to other, original work. Both journals will deal robustly with any confirmed example.

This article was submitted as a duplicate publication to *BMJ Case Reports* and the *Annals* in 2008. On discovery of this in 2012, following the indexing of *BMJ Case Reports*, the article in the *Annals* has been retracted as per the Committee on Publication Ethics guidelines. Manuscripts may not be duplicated. This includes submissions to online as well as paper journals, regardless of indexing. Authors unsure as to whether parts of manuscripts or different aspects of their research may be submitted to multiple journals are advised to contact the editorial teams of the respective journals before submission, to include this in their disclosures and to check carefully the final drafts of the manuscripts before publication online or on paper. Further advice is available at http://publicationethics.org/.
